# Case Report of Myroides odoratimimus Cellulitis in Chronic Venous Stasis Dermatitis With Literature Review

**DOI:** 10.7759/cureus.45319

**Published:** 2023-09-15

**Authors:** Stephen Do, Alka Rebentish, Preethi Ravichandran Kumar

**Affiliations:** 1 Medicine, Touro University Nevada, Henderson, USA

**Keywords:** infectious disease pathology, secondary bacterial infection, multi-drug resistant bacteria, skin and soft tissue, myroides odoratimimus, venous stasis dermatitis

## Abstract

*Myroides *spp.-induced cutaneous infections are rare, with only 17 reported cases in the literature. *Myroides *spp. behave like low-grade opportunistic pathogens, with symptomatic infections observed typically in severely immunocompromised patients and seldom in immunocompetent patients. In this paper, we present an immunocompetent 61-year old male with a past medical history of hypertension, hyperlipidemia, morbid obesity, and patient-reported peripheral neuropathy who presented to the transitional care clinic with bilateral lower extremity swelling and hemosiderin-pigmented dry wounds consistent with diagnosis of chronic venous stasis dermatitis with resolved secondary *Myroides odoratimimus* infection. Further literature review about *Myroides* spp. and its resistance mechanism, antibiotic susceptibility, and biofilm production are also included in this paper.

## Introduction

*Myroides* spp. are aerobic, yellow-pigmented, non-lactose fermenting, oxidase-positive, gram-negative rod bacteria found in the environment [1]. Formerly known to be part of the genus *Flavobacterium* spp. when it was created in 1923, the pathogen has since been reclassified to *Myroides odoratus* and *Myroides odoratimimus* following genotypic and phenotypic analysis by Vancanneyt et al. in 1996 [2].

*Myroides* spp., like other members of the *Flavobacteriaceae* genus, are habit-specific organisms found in soil and water [3]. Since 2010, in the US National Institutes of Health’s National Library of Medicine, reports state that *Myroides odoratus* have been found from hospital effluent [4] and *Myroides odoratimimus* from a pig bite [5] and the gut of grey mullet [6]. A majority of novel species pertaining to the genus of *Myroides* have been reported in the literature, elucidating the genus’ prevalence in nature. They include *M. indicus *and *M. xuanwuensis* from soil, *M. marinus* and *M. pelagicus* from seawater, and lastly, *M. profundi* from deep sea sediments [3]. A minority of novel *Myroides *species, such as *M. phaeus *and *M. injenensis*, have been isolated from human blood, saliva, and urine [7].

Over the last two decades, infections caused by *Myroides* spp. have been increasingly reported in the literature given its resistance to a wide range of antibiotics (including b-lactams and aminoglycosides) making it difficult to treat effectively in the hospital setting [3]. According to Meyer in 2019, of the reported cases that have been noted in the literature, only 16 have been cutaneous infections [8] and if we note a case presented by Foo et al. in 2020 [9], this paper will be presenting the 18th known reported case.

## Case presentation

A 61-year-old male with a past medical history of hypertension, hyperlipidemia, morbid obesity, and patient-reported peripheral neuropathy presented to the transitional care clinic in Las Vegas, Nevada with bilateral lower extremity swelling and hemosiderin-pigmented dry wounds consistent with the diagnosis of chronic venous stasis dermatitis with resolved secondary infection. All of the patient’s history up until his admittance to the hospital and transitional care clinic is self-reported over an eight-month period.

The patient’s symptoms initially presented as sudden discoloration around his ankles bilaterally. He was diagnosed with venous insufficiency by his primary care provider. In the month that followed, the skin surrounding his bilateral lower extremities (BLE) began to ulcerate. At a subsequent urgent care visit, the patient was prescribed his first antibiotic course of clindamycin intravenously for two weeks. His symptoms did not improve, and he was subsequently started on an oral course of trimethoprim-sulfamethoxazole, which also failed to improve his condition. A ClariVein™ procedure was performed on the patient during month five and was successful, as reviewed by both the vascular surgeon and an internist. ClariVein™ is a minimally invasive procedure used to close faulty valves that cause venous pooling. Despite surgical intervention, however, the patient’s leg wounds failed to heal. The patient was then referred to an infectious disease specialist to address the secondary bacterial infection of his venous stasis ulcers. An infectious disease specialist prescribed the patient at-home intravenous infusion of daptomycin once daily and intravenous cefepime twice daily via peripherally inserted central catheter for five weeks and scheduled wound care.

Eight months after the initial presentation and still continuing his intravenous antibiotic regimen of daptomycin and cefepime, the patient made a visit to the emergency department with complaints of unbearable pain and decrease in quality of life. He was admitted during this visit and bilateral lower extremity arterial ultrasound showed no evidence of arterial vessel occlusion. BLE venous ultrasound showed no evidence of deep venous thrombosis (DVT). A surgical culture of the right lower extremity, taken prior to hospitalization, resulted in findings positive for heavy growth of *Myroides odoratimimus*. Susceptibility report for *Myroides odoratimimus* can be seen below in Table 1. The patient continued his treatment with both daptomycin and cefepime intravenously throughout the hospital stay before being discontinued due to the absence of any erythema or open wounds at his bilateral lower extremity. Lab results also showed no evidence of systemic inflammatory response syndrome (SIRS): body temperature between 36-38ºC, heart rate < 90 beats/min, respiratory rate < 20 breaths/min, and WBC count < 12,000 cells/mm^3^. For bilateral lower extremity pain, the patient was prescribed oral duloxetine 60 mg qd and oxycodone/acetaminophen 10 mg/325 mg tablets prn q6 hrs. The patient reported significant improvement of BLE neuropathic pain for 8-10 hours upon taking duloxetine. He declined taking oxycodone/acetaminophen 10 mg/325 mg tablets due to personal reasons. For BLE swelling, the patient was prescribed furosemide 20 mg PO bid and potassium chloride 20 mEq daily.

**Table 1:** Minimal inhibitory concentration report of the patient’s positive Myroides odoratimimus via surgical culture. The patient’s hospital antibiotic course consisted of continuing the patient’s infectious disease specialist’s prescription of IV daptomycin and IV cefepime.

Myroides species	MIC	INTERP
AMIKACIN	> 32	R
AZTREONAM	<=4	S
CEFEPIME	<=2	S
CEFTAZIDIME	<=1	S
CEFTRIAXONE	32	I
CIPROFLOXACIN	2	I
GENTAMICIN	<=2	S
LEVOFLOXACIN	4	I
PIPERACIL/TAZO	<=8	S
TOBRAMYCIN	> 8	R
TRIMETH/SULFA	2/38	S

**Figure 1:**
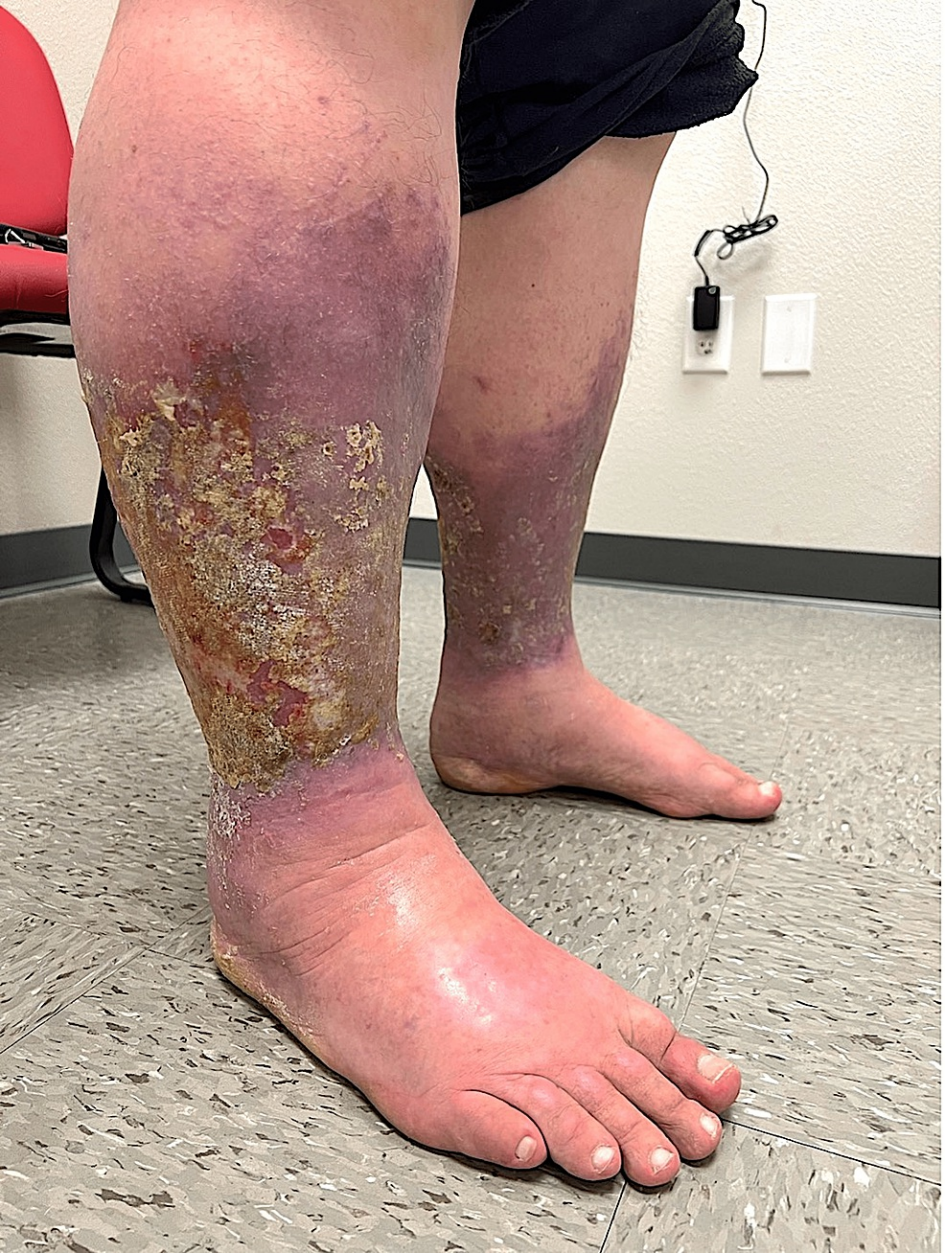
Photograph of chronic venous stasis dermatitis associated with resolved secondary infection from Myroides odoratimimus, taken following discharge from a local hospital and at our transitional care clinic.

**Figure 2:**
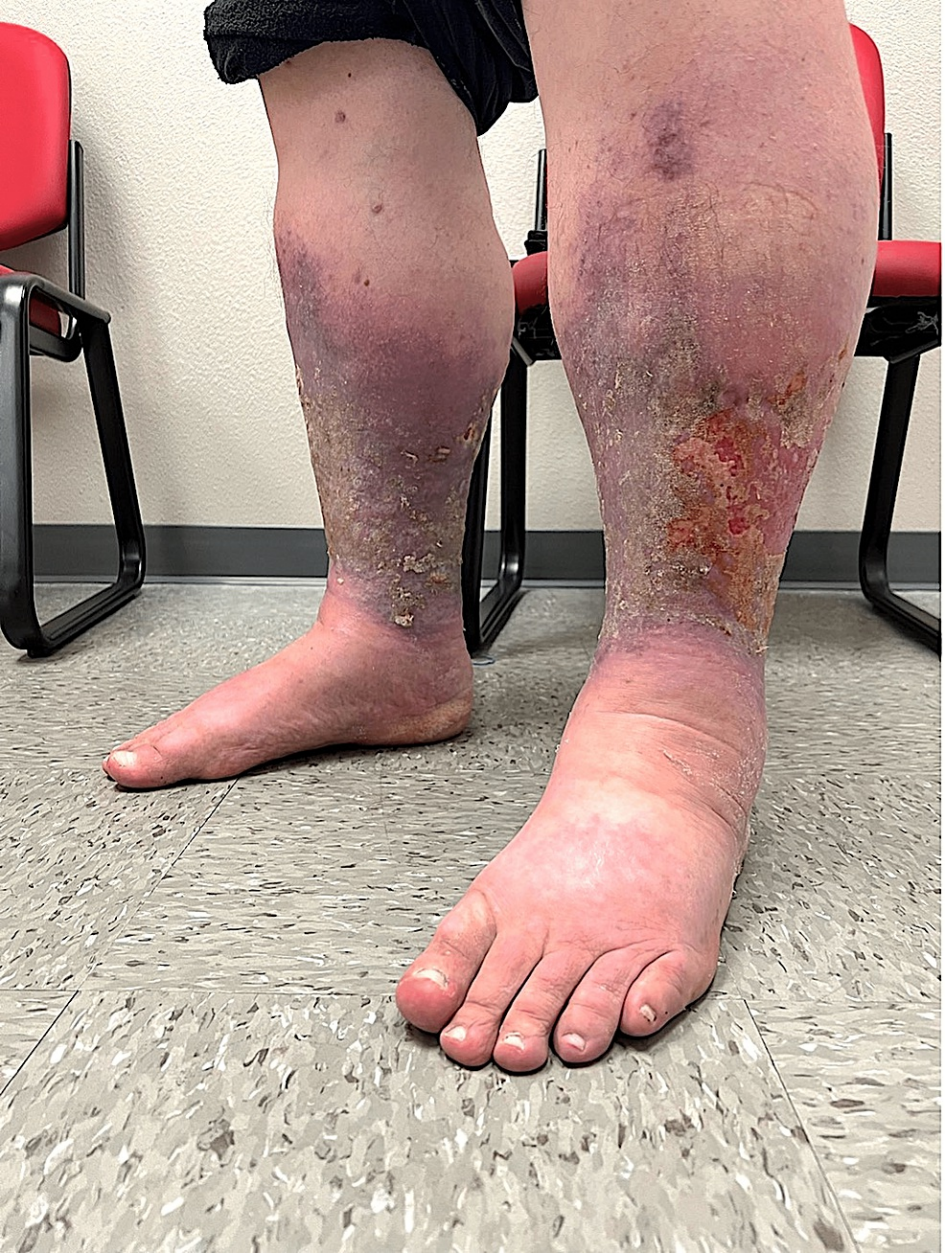
Photograph from another angle of chronic venous stasis dermatitis associated with resolved secondary infection from Myroides odoratimimus, taken following discharge from a local hospital and at our transitional care clinic.

Following discharge, the patient followed up with the hospital visit at our transitional care clinic. In addition to hypertension, hyperlipidemia, morbid obesity, and patient-reported peripheral neuropathy, the patient denied any history of tobacco use, recreational drug use, and alcohol abuse. The patient reported no recent outdoor activities or travels in the last year or having any pets at home. PHQ-9 score of 8. GAD-7 score of 2. Vital signs were 36ºC, heart rate 75 beats/min, blood pressure 106/66 mmHg, respiratory rate 16 beats/min, O2 saturation 94%, and BMI 47.2. Active medications included ascorbic acid 1000 mg qd, atorvastatin 40 mg qd, cholecalciferol 10 mcg, duloxetine 60 mg qd, furosemide 20 mg PO bid, and gabapentin 300 mg qd. Since the onset of his symptoms eight months prior, the patient had lost 13 kg. During this visit, the patient described his bilateral lower extremity pain as “stinging and burning.” The patient endorsed no issues ambulating with tolerable burning pain. Removal of bandages on the patient’s bilateral lower extremity, distal to the knee, showed purple discoloration with healing 3x3 cm ulcers (left worse than right) evidenced by peeling skin, and bilateral foot edema (Figures 1, 2). The patient’s BLEs exhibited only extreme pain to light touch and moderate serous exudate. His bilateral lower extremity did not have any greater warmth or redness, frank purulence, and lymphangitis outside the borders of stasis dermatitis. No gross motor deficits, strength 5/5 bilaterally, sensation grossly intact throughout to light touch, and deep tendon reflexes 2+ symmetrically, on exam.

At the conclusion of this transitional care clinic visit, the patient was instructed to continue furosemide 20 mg PO bid and potassium chloride supplementation to continue reducing his bilateral lower extremity swelling. He was referred to follow up with an infectious disease specialist to assess the need for any additional courses of antibiotics and long-term follow-up.

## Discussion

*Myroides* spp. cutaneous infections have only ever been reported in 17 previous cases in the literature. Of the reported cases of infections caused by *Myroides* spp. [5, 8-24], cutaneous infections have been the most common. Sources of such cutaneous infections have been from traumatic injury to the left arm by a cutter bar in a farming accident [10], farming barefoot [12], pig bite [5], walking barefoot through heavily wooded area, or dog lick [8], gardening barefoot [9], and whitewater rafting or mineral spa [17]. When asked, our patient denied any recent travels, significant outdoor activities, or pets at home to prove a possible mechanism of inoculation from these reported etiologies. Aside from these environmental sources, other possible culprits of inoculation that can be attributed to our patient are sewage water during a house flood [16], hospital effluent [4], and iatrogenic contamination as shown in hospital outbreaks [23]. Given what is currently reported in the literature for *Myroides* spp. infection and based on the patient’s history of frequent visits to medical facilities throughout the eight months of his illness, house sewage and hospital-acquired infection seem to be the most likely causes. Further research may involve sampling the patient’s water supply at home and inventorying of patients who have positive wound cultures for *Myroides* spp. throughout Las Vegas medical facilities.

*Myroides* spp. has a known history of being difficult to treat because of its multidrug resistance. In the literature, two chromosome-encoded b-lactamases, MUS-1 and TUS-1, have been identified as resistance mechanisms in *Myroides odoratimimus* and *Myroides odoratus* [25]. In addition, MUS-2, a novel variant of chromosome-encoded b-lactamase MUS-1, was isolated from livestock animals in Lebanon adding to the limited list of what is known thus far about the pathogen’s virulence mechanism [26]. From Mammeri et al. study on TUS-1 and MUS-1, they found that *Myroides odoratimimus* showed reduced susceptibility to cefoxitin, cefepime, cefpirome, imipenem, meropenem and complete resistance to amoxicillin, ticarcillin, piperacillin, cephalothin, moxalactam, cefotaxime, cefuroxime, ceftazidime, and aztreonam [25]. More studies have been done to attempt to elucidate other virulence factors of *Myroides* spp., but the exact pathophysiological mechanisms require more understanding. In a study that analyzed the complete genome sequence of a PR63039 strain of *M. odoratimimus*, Ming et al. found that the genome consisted of one chromosome and one plasmid [27]. Resistance gene distribution analysis further suggested that there was a resistance region in the genome, and prophage structure analysis hinted at multiple resistance-inducing gene mutations that contribute to the diverse antibiotic resistance mechanisms of the PR63039 strain [27].

Despite the limited understanding of the pathogenicity of *Myroides* spp., the pathogen has been successfully treated in skin and soft tissue infections (SSTIs) based on minimal inhibitory concentration (MIC) reports with antibiotics such as piperacillin/tazobactam and teicoplanin [10], ciprofloxacin alone [5, 12], ciprofloxacin with imipenem and cilastatin [11], trimethoprim-sulfamethoxazole alone [13], trimethoprim-sulfamethoxazole and ciprofloxacin [14], meropenem alone [8, 15, 17, 18], and ampicillin-sulbactam alone [16]. A comprehensive study of 20 antibiotics against 59 strains of *Myroides odoratimimus* and *Myroides odoratus* (43 *M. odoratimimus* and 16 *M. odoratus*) was done by Gunzer et al. in 2018, elucidating a better understanding of the pathogen’s susceptibilities [3]. The study revealed that only a majority of strains showed intermediate susceptibility to ampicillin than to piperacillin/tazobactam [3]. Moreover, no strain was susceptible to ceftazidime, cefepime, and aztreonam. Meropenem was shown to be more effective for the treatment of *Myroides* infections than imipenem. Of the quinolones tested in the Gunzer et al. study, moxifloxacin showed the lowest MIC values in its antibiotic class [3]. Based on high MIC values, Gunzer et al. assumed that *M. odoratimimus* and *M. odoratus* were naturally resistant to colistin, fosfomycin, daptomycin, gentamicin, and amikacin [3]. As for trimethoprim/sulfamethoxazole, erythromycin, and rifampicin, MIC results varied from low to high levels. The results from this study are relatively consistent with successful treatments of SSTIs caused by *Myroides* spp. in literature and may prove useful for healthcare providers who may encounter this pathogen in practice. However, it is worth noting that the MIC report received from our patient’s isolate showed contrasting antibiotics susceptibilities than the results from isolates studied by Gunzer et al. [3]. Gunzer et al.’s study revealed no susceptibility to ceftazidime, cefepime, aztreonam, and gentamicin, while our patient’s MIC report had susceptibility values to all of the above [3]. It is possible that our patient’s *Myroides odoratimimus* strain was not part of Gunzer et al.’s study and healthcare practitioners should treat based on MIC obtained [3]. This is supported by the fact that the patient had no signs and symptoms of active *Myroides odoratimimus* infection by the time of discharge from the hospital and presentation to our transitional care clinic after being treated with intravenous daptomycin and cefepime.

The timeline of when our patient’s BLE venous stasis became infected with *Myroides odoratimimus* is not clear, but the chronicity of his disease lends credence to the finding of *Myroides odoratimimus*’s ability to form biofilms [28]. Pompilio et al. were able to evaluate the biofilm-forming ability of *M. odoratimimus* using an *in-vitro* “skin-like” model and “simulated wound fluid” to mimic the serous exudate [28]. The results from the study indicated that *M. odoratimimus* biofilm is significantly more resistant to all tested antibiotics, compared with its planktonic counterpart. Of the antibiotics tested against them, none were able to eradicate the biofilm, even at maximum bactericidal concentrations. Tigecycline did not result in a significant vitality reduction and meropenem and levofloxacin were able to achieve high significant reduction in biofilm vitality. *In-vivo*, two studies have identified the ability of *Myroides odoratimimus *to form biofilm in humans, causing urinary tract infection (UTI) [22] and infection to a recurrent calcaneal ulcer [28]. In the study of *Myroides odoratimimus*-induced recurrent calcaneal ulcer, the strain was unable to reach the bloodstream. This helped postulate the pathogen’s low invasive potential. However, it was able to form a high amount of biofilm, with increasing capability over time, suggesting a possible role in chronic infection [28]. To counteract biofilm forms of *M. odoratimimus*, researchers have already begun investigating combination forms of antibiotic treatment with success in colistin/ciprofloxacin and meropenem/ciprofloxacin combinations [29]. MALDI-TOF MS has been proposed to be a better suitable tool in helping identify *Myroides* at the species level [30].

As a limiting factor, we were not able to obtain a majority of the patient’s medical history until the eighth month of his disease course when he was seen by us at the transitional care clinic. This patient may have had other diagnostic tests done during this period that may elucidate more information regarding this disease, such as the timeline of when his infection was the most severe. Given these limitations, we are only able to offer a snapshot of *Myroides odoratimimus* infection secondary to venous stasis dermatitis toward its resolution. We followed up with our patient over the phone six months after his visit and he noted no recurrence.

## Conclusions

*Myroides* spp. infections have been increasingly reported in the last two decades, with cellulitis being the most common. This is the 18th reported case of *Myroides* spp.-induced cutaneous infection. There is still much to be learned about its multidrug resistance, susceptibility, pathogenic patterns, and effect on immunocompetent hosts, which previously were believed to affect only immunocompromised patients. We report this patient case to raise awareness of this pathogen and we implore others to report their own cases attributed to *Myroides* spp. in hopes of improving the quality of patient care and outcomes.
